# CCL18 as a Biomarker of Interstitial Lung Disease (ILD) and Progressive Fibrosing ILD in Patients with Idiopathic Inflammatory Myopathies

**DOI:** 10.3390/diagnostics13101715

**Published:** 2023-05-12

**Authors:** Elisabetta Zanatta, Andrea Martini, Roberto Depascale, Anna Gamba, Marta Tonello, Mariele Gatto, Chiara Giraudo, Elisabetta Balestro, Andrea Doria, Luca Iaccarino

**Affiliations:** 1 Rheumatology Unit, Department of Medicine, University Hospital of Padova, 35128 Padova, Italy; elisabetta.zanatta@unipd.it (E.Z.); roberto.depascale@studenti.unipd.it (R.D.); anna.gamba@studenti.unipd.it (A.G.); marta.tonello@unipd.it (M.T.); mariele.gatto@unipd.it (M.G.); luca.iaccarino@unipd.it (L.I.); 2 Unit of Internal Medicine and Hepatology (UIMH), Department of Medicine, University Hospital of Padova, 35128 Padova, Italy; andrea.martini@aopd.veneto.it; 3 Unit of Advanced Clinical and Translational Imaging, Department of Medicine—DIMED, Padova University Hospital, 35128 Padova, Italy; chiara.giraudo@unipd.it; 4 Respiratory Disease Unit, Department of Cardiac, Thoracic, Vascular Sciences and Public Health, Padova University Hospital, 35128 Padova, Italy; elisabetta.balestro@aopd.veneto.it

**Keywords:** interstitial lung disease (ILD), idiopathic inflammatory myopathies (IIMs), biomarkers, autoantibodies, progressive pulmonary fibrosis

## Abstract

Objectives. To assess CCL18 and OX40L as biomarkers of interstitial lung disease (ILD) and/or progressive fibrosing (PF-) ILD in idiopathic inflammatory myopathies (IIMs). Methods. Patients with IIMs seen in our center from July 2020 to March 2021 were consecutively enrolled. ILD was detected by high-resolution CT. CCL18 and OX40L serum levels were measured by validated ELISA assays in 93 patients and 35 controls. At the 2-year follow-up, PF-ILD was evaluated according to the INBUILD criteria. Results. ILD was diagnosed in 50 (53.7%) patients. CCL18 serum levels were higher in IIMs patients vs. controls (232.9 [IQR 134.7–399.07] vs. 48.4 [29.9–147.5], *p* < 0.0001), with no difference for OX40L. IIMs-ILD patients exhibited higher levels of CCL18 than those without ILD (306.8 [190.8–520.5] vs. 162 [75.4–255.8], *p* < 0.0001). High CCL18 serum levels were independently associated with IIMs-ILD diagnosis. At follow-up, 22/50 (44%) patients developed a PF-ILD. Patients who developed PF-ILD had higher CCL18 serum levels than non-progressors (511 [307–958.7] vs. 207.1 [149.3–381.7], *p* < 0.0001). Multivariate logistic regression analysis revealed CCL18 as the only independent predictor of PF-ILD (OR 1.006 [1.002–1.011], *p* = 0.005). Conclusions. Although in a relatively small sample, our data suggest that CCL18 is a useful biomarker in IIMs-ILD, particularly in the early identification of patients at risk of developing PF-ILD.

## 1. Introduction

Interstitial lung disease (ILD) is the most frequent organ involvement in patients with idiopathic inflammatory myopathies (IIMs) and is detectable in about 50% of cases, though shared screening strategies are still lacking [1]. Moreover, ILD is characterized by a highly variable course in connective tissue diseases (CTDs), ranging from mild and stable to progressive life-threatening forms [2,3]. The term progressive fibrosing (PF-)ILD encompasses a group of pulmonary diseases of various origins which can progress despite treatment [3]. Given the recent development of new treatments for lung fibrosis, the early identification of CTD patients who are more likely to develop PF-ILD is critical. Data from registries have recently shown that PFD-ILD occurred in a consistent proportion of IIMs patients (about 40% according to the INBUILD criteria) [4]. Nevertheless, the optimal monitoring strategies for IIMs-ILD have not been defined yet [5], due to several concerns regarding the identification of patients at-risk of progression, the costs and ionizing radiation exposure of repeating chest high-resolution computed tomography (HRCT).

The chemokine CCL18, previously known as pulmonary and activation-regulated chemokine, is constitutively expressed by antigen-presenting cells—particularly dendritic cells and macrophages in lung tissues—and is highly inducible by inflammatory stimuli [6]. It has been shown that CCL18, likely via its cognate receptor CCR8, may induce collagen synthesis in lung fibroblasts and thereby contribute to fibrosis and subsequent deterioration of lung function [7]. Circulating CCL18 serum levels correlated with the severity of fibrosis in idiopathic pulmonary fibrosis (IPF) [8] and can predict ILD progression in systemic sclerosis (SSc) [9,10]. OX40 ligand (OX40L) is a glycoprotein expressed on activated antigen-presenting cells and bound to the tumor necrosis factor receptor. Both OX40L and soluble OX40L were overexpressed in several autoimmune diseases, such as systemic lupus erythematosus and SSc [11,12]. In vivo OX40L blockade prevented inflammation-driven fibrosis of the skin, lung and vessels in different complementary mouse models of SSc [13]. In patients with IIMs, OX40 and OX40L were overexpressed by different types of cells (i.e., T cells, macrophages, and B cells) within limb muscle specimens [14].

We aimed to assess the potential role of CCL18 and OX40L as biomarkers of ILD and/or PF-ILD in patients with IIMs.

## 2. Materials and Methods

### 2.1. Study Population

Patients affected with IIMs—according to Bohan and Peter criteria [15], ENMC criteria [16] or 2017 EULAR/ACR classification criteria [17]—aged >18 years who attended our referral center between July 2020 and March 2021 were consecutively enrolled and followed up over a 2-year period. Patients affected with group 1 pulmonary arterial hypertension and/or chronic obstructive lung diseases were excluded. Thirty-five healthy volunteers (matched for age and sex) were also included as controls. The study was approved by our institution’s Ethics committee (Azienda Ospedaliera di Padova, Padova, Italy, 5505/AO/22), and all participants provided written informed consent.

### 2.2. Data Collection from IIMs Patients

Demographic, clinical, and serological variables were recorded for each patient. All patients had at least one chest HRCT and one assessment of pulmonary function tests (PFTs) performed within the 6 months before the enrollment. ILD was diagnosed by HRCT in the presence of reticular abnormalities, ground-glass opacities and/or honeycombing. Three patterns of ILD were identified: usual interstitial pneumonia (UIP), nonspecific interstitial pneumonia (NSIP) and organizing pneumonia (OP) [18].

Serum anti-nuclear antibodies (ANA) were analyzed by immunofluorescence (IF) assay on HEp-2 cells, anti-extractable nuclear antigen (ENA) antibodies by enzyme-linked immunosorbent assay (ELISA) and immunoblot, MSA and MAA by commercial line blots (Euroline Myositis Profile, Euroimmun, Lubeck, Germany) including recombinant human proteins for Mi-2 alpha, transcription intermediary factor 1-gamma (TIF1γ), small ubiquitin-like modifier-1 activating enzyme (SAE), Ku, PM-Scl75/100, MDA-5, signal recognition particle (SRP), Jo-1, PL-7, PL-12, EJ and OJ.

At baseline, the following PFTs indices (expressed as the percentage of observed/theoretic values) were recorded: forced vital capacity (FVC), total lung capacity (TLC), diffusing capacity for carbon monoxide (DLCO) and carbon monoxide transfer coefficient (KCO) calculated as the ratio between DLCO and alveolar volume (VA). 

At 24 months, we retook PFTs and HRCT, and collected the following data: worsening of respiratory symptoms (i.e., dyspnea and cough), new requirement or increased need for supplemental oxygen, new onset pulmonary hypertension and ongoing immunosuppressive and glucocorticoids therapy. According to the INBUILD CRITERIA [19], PF-ILD was defined as FVC decline ≥10% or a relative FVC decline ≥5 and <10% in the presence of worsening of respiratory symptoms or fibrosis extent on HRCT or worsening of respiratory symptoms in presence of fibrosis extent, all within 24 months from the baseline evaluation. In the presence of worsening dyspnea, other causes were excluded (i.e., by performing EKG and Echocardiography to exclude a cardiogenic origin).

### 2.3. Quantitative Analyses of Candidate Serum Biomarkers

Blood samples were collected during routine blood tests, using standardized procedures and processing: centrifugation at 3000 rpm for 10 min to separate the supernatant; serum samples were stored at −80 °C until assayed. Serum PARC/CCL18 was analyzed by enzyme immunoassay (Human PARC/CCL18 ELISA Kit, Invitrogen, Thermo Fischer Scientific, Waltham, MA, USA). According to the experimental protocol, serum samples were assayed in duplicate using a 1:1000 dilution. The optical density was measured in a microtiter plate reader at 450 nm using the TECAN Sunrise III (Tecan, Männedorf, CH, Switzerland). The concentrations, expressed as ng/mL, were calculated using the standard curves generated according to specific standards provided by the manufacturer. The enzyme immunoassay (Human OX40L ELISA Kit, Invitrogen, Thermo Fischer Scientific) was used to measure serum levels of human OX40L. In agreement with the experimental protocol, serum samples were assayed in duplicate using a 1:1 dilution. The concentrations, expressed as ng/mL, were calculated using the standard curves generated according to specific standards provided by the manufacturer. The intra- and inter-assay coefficients of variation were <10% for both assays. Serum samples from 35 healthy controls were also analyzed.

### 2.4. Statistical Analysis

Continuous variables were expressed as median (interquartile range) and categorical variables as frequency and percentage. Comparison between groups (i.e., IIMs-ILD vs. no ILD, and progressors vs. non progressors) was carried out using the Mann–Whitney U test for continuous variables and the chi-squared test or Fisher’s exact probability test for categorical data, where appropriate. The ability of CCL18 to diagnose IIMs-ILD and to identify patients who may develop PF-ILD was assessed by receiver operating characteristic (ROC) curve analysis. Sensitivity, specificity, positive predictive value (PPV) and negative predictive value (NPV) were calculated. To avoid collinearity, two multivariate models were performed to identify factors independently associated with the diagnosis of ILD by logistic regression. Logistic regression was also performed to determine independent predictors of PF-ILD. Variables found to be different (*p* < 0.1) at univariate analysis were included in the multivariate logistic regression models (with backward elimination), adjusted for age and sex. All tests were two-tailed, and *p* values <0.05 were considered significant. The statistical analysis was performed using the SPSS statistical package, version 22.0.

## 3. Results

### 3.1. Study Population

We enrolled in the study 93 IIMs patients: 61% women; the mean age range was 62 (54.5–71) years, and mean disease duration was 3 (2–9) years. Baseline HRCT showed findings consistent with lung fibrosis in 50/93 (53.7%) patients. Demographic, clinical, serological, and functional features of patients with and without ILD are reported in Table 1.

The two groups of patients were similar in terms of demographic features and disease duration. IIMs patients affected with ILD more frequently had positive myositis specific and associated autoantibodies, including antisynthetase antibodies as compared to those without ILD. Mechanic’s hands (*p* = 0.013) and dyspnoea (*p* < 0.0001) were more common, and arthritis (*p* = 0.058) tended to be more common in the IIMs-ILD group than in patients without ILD. As expected, lung volume indices (i.e., FVC and TLC) and DLCO were lower (more compromised) in patients with ILD than in those without; mycophenolate mofetil was administered more frequently to patients with ILD (*p* = 0.027), and methotrexate was administered to those without ILD (*p* = 0.001) (Table 1). Among patients with IIMs-ILD, 32 (64%) had NSIP pattern; 8 (16%) had UIP pattern, and 10 (20%) had OP pattern on HRCT. 

#### 3.1.1. Performance of Serum Markers for the Diagnosis of IIMs-ILD

**Figure 1:**
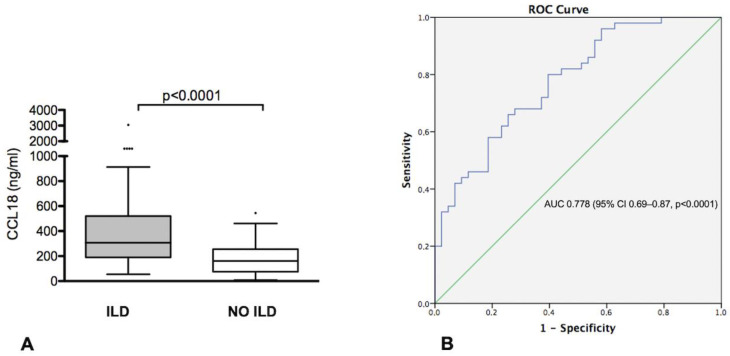
(**A**) CCL18 serum levels in idiopathic inflammatory myopathies (IIMs) patients with and without interstitial lung disease (ILD); (**B**) ROC curve illustrating the diagnostic value of CCL18 for diagnosis of ILD in patients with IIMs.

OX40L serum levels were similar between patients and controls (*p* = 0.971), whereas CCL18 serum levels were higher in IIMs patients vs. controls (232.9 [IQR 134.7–399.07] vs. 48.4 [IQR 29.9–147.5] ng/mL, *p* < 0.0001). Among IIMs patients, those with ILD had higher levels of CCL18 than those without (306.8 [190.8–520.5] vs. 162 [75.4–255.8] ng/mL, *p* < 0.0001, Figure 1A).

ROC curve analysis to assess the performance of CCL18 in identifying IIMs-ILD showed an area under the curve (AUC) of 0.778 (95% CI 0.69–0.87, *p* < 0.0001) (Figure 1B). Using a threshold of 234.7 ng/mL, defined by the ROC curve, CCL18 showed a 68% sensitivity, 72% specificity, 74% PPV and 66% NPV in identifying IIMs-ILD in our cohort.

#### 3.1.2. Multivariate Analysis for the Diagnosis of IIMs-ILD

We performed two models of multivariate analysis (Table 2). In Model 1, lower values of TLC (OR 0.944 [95% CI 0.900–0.991], *p* = 0.019), high CCL18 serum levels (OR 1.014 [95% CI 1.005–1.023, *p* = 0.001]) and the clinical phenotype (OR 4.949 [95% CI 1.516–16.103], *p* = 0.008) were independent predictors of PF-ILD. The only independent predictors of lung fibrosis progression in Model 2 were high CCL18 serum levels (OR 1.009 [95% CI 1.003–1.015], *p* = 0.004) and positive antisynthetase autoantibodies (OR 5.075 [95% CI 1.120–22.999], *p* = 0.035).

#### 3.1.3. Comparison between Patients with or without a PF-ILD

**Figure 2:**
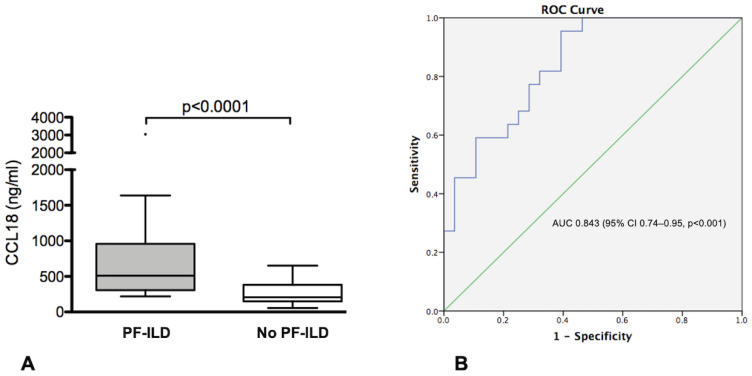
(**A**) CCL18 serum levels in IIMs-ILD patients with and without progressive fibrosing ILD (PF-ILD) (**B**) ROC curve illustrating the value of CCL18 in predicting the occurrence of PF-ILD in patients with IIMs.

At the 24-month follow-up visit, 22/50 (44%) patients had developed PF-ILD. Demographic, clinical, serological, and functional-radiological features of patients with and without PF-ILD are reported in Table 3. At baseline, patients who developed PF-ILD more frequently had positive anti-PL-7 or anti-PL-12 antibodies (*p* = 0.017) and tended to have a lower prevalence of arthritis (*p* = 0.09) and more frequently dyspnea (*p* = 0.071) than non-progressors. No other baseline differences regarding autoantibody profile, clinical and radiological variables, functional pulmonary features, and treatment were found in progressors vs. non-progressors. Patients who developed PF-ILD had higher serum levels of CCL18 at baseline than non-progressors (511 [307–958.7] vs. 207.1 [149.3–381.7] ng/mL, *p* < 0.0001) (Figure 2A).

ROC curve analysis to assess the performance of CCL18 in identifying patients with PF-ILD showed an area under the curve (AUC) of 0.843 (95% CI 0.74–0.95, *p* < 0.001) (Figure 2B). Using a threshold of 303.5 ng/mL, defined by the ROC curve, CCL18 showed an 82% sensitivity, 69% specificity, 67% PPV and 83% NPV in predicting the occurrence of PF-ILD in our cohort.

#### 3.1.4. Multivariate Analysis for the Occurrence of PF-ILD

Multivariate analysis for the occurrence of PF-ILD showed CCL18 as the only independent predictor of PF-ILD (OR 1.007 [1.002–1.011], *p* = 0.008) (Table 4).

## 4. Discussion

The recent development and approval of new treatments for CTD-ILD has highlighted the urgent need to identify reliable predictors of PF-ILD occurrence, to early stratify patients and optimize their management [2]. The evaluation of some disease features may not be univocal among physicians, especially in non-referral centers [20]. Hence, the emergence of serum biomarkers—objectively measurable indicators of physiological or pathological processes—as promising diagnostic and prognostic tools in clinical practice [10,21].

We found that increased levels of CCL18 can independently predict the development of PF-ILD in patients with IIMs-ILD. In fact, our findings and particularly the ROC curve suggest that CCL18 may better identify patients likely to develop PF-ILD, rather than the presence of ILD itself. This ability of the chemokine seems particularly intriguing in clinical practice, since screening methods for ILD have improved in recent years while early identification of patients at risk for PF-ILD is still one of the main challenges.

CCL18 has been found increased in serum, bronchoalveolar lavage fluid or sputum of patients with T-helper 2 (Th2)-predominant diseases such as IPF, hypersensitivity pneumonia and Sc [22]. In patients with IIMs, the immune response is characterized by an increase of the percentage of Th1 and Th2 lymphocytes in the serum, with higher Th2/Th1 and Th2/Th17 ratios vs. controls [23,24]. It has been demonstrated that increased CCL18 levels are predictive of mortality and pulmonary fibrosis progression in patients with IPF and SSc [8,25]. Although ILD progression has been more thoroughly investigated in Sc [26] than in other CTDs, a very recent report from the Canadian ILD registry has highlighted that about 40% of IIMs-ILD patients may develop PF-ILD according to the INBUILD criteria, despite treatment [4]. The percentage was similar in our study population, indicating that IIM-ILD patients should be carefully monitored for ILD progression. Among several clinical and serological IIM-specific features and ILD-specific characteristics (i.e., radiological pattern and PFTs impairment), CCL18 emerged as the only independent predictor of PF-ILD in our cohort. Moreover, the ROC curve analysis allowed us to identify 301 U/mL as the optimal threshold for the prediction of PF-ILD in IIMs patients, with a sensitivity and VPN above 80%. Thus, our findings suggest that the evaluation of CCL18 at baseline could help stratifying the risk of IIMs patients with ILD leading to a close monitoring of patients at higher risk to develop PF-ILD by reassessing PFTs and HRCT to the optimization of patients’ management (e.g., adopting a more aggressive therapeutic strategy in earlier stages of IIMs-ILD). In this regard, it bears noting the lack of shared strategies on ILD monitoring in IIMs, along with the absence of a formal international consensus on treatment of this frequent and potentially life-threatening organ involvement in IIMs.

It should be noted that in our study the mean values of CCL18 in IIMs-ILD patients are considerably higher than those reported in Rheumatoid Arthritis [27] and SSc [10] and closer to those reported in active hypersensitivity pneumonia [22]. This could be due to the more inflammatory nature of IIMs-ILD compared for example with SSc-ILD, as CCL18 is known to be highly inducible by inflammatory stimuli.

As a limitation of our study, the relatively small sample size may have underpowered some differences; e.g., although 4/6 IIMs-ILD patients with anti-MDA5 positivity developed PF-ILD, the data were not statistically significant. Nevertheless, it must be considered that IIMs are recognized as a rare disease. Moreover, the lack of a radiological score evaluation did not allow to define the extent of ILD, in addition to functional impairment. By contrast, one strength of our study is that all patients were enrolled from a single, homogeneous, and well-characterized cohort of IIMs.

In conclusion, CCL18—but not OX40L—may be a useful diagnostic biomarker in the assessment of patients with IIMs-ILD, and it seems to be particularly promising in the early identification of the PF-ILD forms. Larger studies are needed to assess its clinical applicability and ascertain its sensitivity to changes, since only baseline serum levels were evaluated in our cohort. Furthermore, the potential predictive value of monitoring the clinical response to treatment (i.e., immunosuppressants and/or antifibrotics) should be assessed.

## Figures and Tables

**Table 1:** Demographic, serological and clinical features in all patients and according to the presence of ILD.

	All Patients (*n* = 93)	IIMs-ILD (*n* = 50)	IIMs without ILD (*n* = 43)	*p*
Age, yrs	62 (54.5–71)	62.5 (57–71)	62 (49–71)	0.284
Female Sex	61 (65.6)	31 (62)	30 (69.8)	0.432
Disease duration, yrs	3 (2–9)	4 (2–11)	3 (2–9)	0.186
DM, *n* (%)	34 (36.6)	11 (22)	23 (53.5)	<0.0001
PM, *n* (%)	30 (32.3)	12 (24)	18 (41.9)	
ASyS, *n* (%)	29 (31.2)	27 (54)	2 (4.7)	
Fever, *n* (%)	5 (5.4)	4 (8.2)	1 (2.3)	0.218
Weight loss, *n* (%)	5 (5.4)	2 (4.1)	3 (7)	0.541
Muscular weakness, *n* (%)	43 (46.2)	23 (46)	20 (46.5)	0.961
MMT-8	146 (140–150)	148 (143.5–150)	145 (140–150)	0.363
Dysphagia, *n* (%)	14 (15.2)	6 (12.2)	8 (18.6)	0.397
Heliotropic Rash, *n* (%)	21 (22.8)	8 (16.3)	13 (30.2)	0.113
Mechanic’s hands, *n* (%)	13 (14.3)	11 (22.9)	2 (4.7)	0.013
Arthritis, *n* (%)	21 (22.8)	15 (30.6)	6 (14)	0.058
Myocarditis, *n* (%)	4 (4.7)	2 (4.4)	2 (4.9)	0.924
PH (group 3), *n* (%)	1 (1.1)	1 (2)	0 (0)	0.351
Dyspnea, *n* (%)	25 (27.2)	22 (44.9)	3 (7)	<0.0001
Cough, *n* (%)	10 (10.9)	7 (14.3)	3 (7)	0.261
FVC	96 (74–100)	89 (70–109)	106.5 (93–114)	0.050
TLC	83 (70–96)	81 (66–92)	92.5 (66–107)	0.032
DLCO	73.5 (62–81.7)	69 (56.5–80)	80 (69–93)	0.013
KCO	86 (73.7–102.7)	86 (67–101)	86 (75–105)	0.510
CK, U/L	100 (64.5–333.5)	100 (50–293)	108 (88–529.5)	0.666
CCL18, ng/mL	232.9 (134.7–399.1)	306.8(190.8–520.5)	162 (75.4–255.8)	<0.0001
ANA, *n* (%)	58 (63)	34 (69.4)	24 (55.8)	0.178
Anti-ENA, *n* (%)	47 (50.5)	34 (68)	13 (30.2)	<0.0001
Myositis-specific antibodies, *n* (%)	60 (64.5)	40 (80)	20 (46.5)	0.001
Myositis-associated antibodies, *n* (%)	41 (44.6)	28 (57.1)	13 (30.2)	0.01
Anti-synthetase antibodies, *n* (%)	32 (34.4)	28 (56)	4 (9.3)	<0.0001
Anti-SSA, *n* (%)	33 (35.5)	22 (44)	11 (25.6)	0.06
Anti-Ro52, *n* (%)	28 (30.1)	20 (40)	8 (18.6)	0.025
Anti-SSB, *n* (%)	3 (3.2)	3 (6)	0 (0)	0.103
Anti-Mi2, *n* (%)	9 (9.7)	2 (4)	7 (16.3)	0.046
Anti-U1RNP, *n* (%)	3 (3.2)	2 (4)	1 (2.3)	0.649
Anti-MDA5, *n* (%)	10 (10.8)	6 (12)	4 (9.3)	0.675
Anti-Jo1, *n* (%)	20 (21.5)	17 (34)	3 (7)	0.002
Anti-PL12, *n* (%)	5 (5.4)	5 (10)	0 (0)	0.033
Anti-PL7, *n* (%)	3 (3.2)	2 (4)	1 (2.3)	0.649
Anti-PL12 or PL7, *n* (%)	8 (7)	7 (14)	1 (2.3)	0.045
Anti-EJ, *n* (%)	3 (3.2)	3 (6)	0 (0)	0.103
Anti-Ku, *n* (%)	3 (3.2)	2 (4)	1 (2.3)	0.649
Anti-NXP2, *n* (%)	3 (3.2)	3 (6)	0 (0)	0.103
Anti-TIF1γ, *n* (%)	6 (6.5)	2 (4)	4 (9.3)	0.299
Anti-SRP, *n* (%)	3 (3.2)	2 (4)	1 (2.3)	0.649
Anti-PMScl, *n* (%)	6 (6.5)	3 (6)	3 (7)	0.848
Glucocorticoids, *n* (%)	62 (70.5)	35 (72.9)	27 (67.5)	0.579
Immunosuppressants, *n* (%)	65 (71.4)	25 (71.4)	30 (71.4)	1.000
Mycophenolate, *n* (%)	27 (34.2)	19 (45.2)	8 (21.6)	0.027
Methotrexate, *n* (%)	32 (40.5)	10 (23.8)	22 (59.5)	0.001
Azathioprine, *n* (%)	3 (3.8)	3 (7.1)	0 (0)	0.097
Rituximab, *n* (%)	3 (3.8)	2 (4.8)	1 (2.7)	0.633

Values are expressed as numbers and (%) or median and Q1–Q3 as appropriate. ANA, anti-nuclear antibodies; ASyS, antisynthetase syndrome; CK, creatine kinase; DLCO, diffusion lung CO; DM, dermatomyositis; ENA, extractable nuclear antigen; FVC, forced vital capacity; ILD, interstitial lung disease; KCO, carbon monoxide transfer coefficient; MDA5, anti-melanoma differentiation-associated gene; MMT, manual muscle test; PH, pulmonary hypertension; PM, polymyositis; RNP, ribonucleoprotein; SRP, signal recognition particle; TIF1γ, transcription intermediary factor 1-gamma; TLC, total lung capacity.

**Table 2:** Multivariate analysis for IIMs-ILD diagnosis.

Variable	OR (CI 95%)	*p*
Model 1		
Disease phenotype (DM/PM/ASyS)	4.949 (1.516–16.103)	0.008
Anti-Ro52	2.408 (0.408–15.060)	0.324
Anti-Mi2	0.128 (0.000–57.47)	0.578
CCL18 serum levels (ng/mL)	1.014 (1.005–1.023)	0.001
Arthritis	0.591 (0.062–5.674)	0.649
TLC	0.944 (0.900–0.991)	0.019
Model 2		
Anti-synthetase antibodies	5.075 (1.120–22.999)	0.035
Anti-Ro52	2.950 (0.656–13.264)	0.158
Anti-Mi2	1.373 (0.048–39.521)	0.853
CCL18 serum levels (ng/mL)	1.009 (1.003–1.015)	0.004
Mechanic’s hands	4.722 (0.442–50.446)	0.199
FVC	0.972 (0.942–1.003)	0.077

Values are expressed as OR (95% CI). CI, confidence interval. ASyS, anti-synthetase syndrome; DM, dermatomyositis; PM, polymyositis; FVC, forced vital capacity; TLC, total lung capacity.

**Table 3:** Demographic, serological, and clinical features in IIMs-ILD progressors vs. non progressors.

	Progressors (*n* = 22)	Non Progressors (*n* = 28)	*p*
Age, yrs	62 (56–72.7)	62.5 (57.5–69.7)	0.922
Female Sex, *n* (%)	16 (72.3)	15 (53.6)	0.166
Disease duration, yrs	4 (2.2–10.7)	3.5 (2–11.7)	0.983
DM, *n* (%)	3 (13.6)	8 (28.6)	0.446
PM, *n* (%)	6 (27.3)	6 (21.4)	
ASyS, *n* (%)	13 (59.1)	14 (50)	
Fever, *n* (%)	1 (4.5)	3 (10.7)	0.404
Weight loss, *n* (%)	0 (0)	2 (7.1)	0.192
Muscle weakness, *n* (%)	8 (36.4)	15 (53.6)	0.226
MMT-8	150 (145–150)	147 (133–150)	0.159
Dysphagia, *n* (%)	1 (4.5)	5 (17.8)	0.138
Heliotropic Rash, *n* (%)	2 (9.1)	6 (21.4)	0.216
Mechanic’s hands, *n* (%)	3 (13.6)	8 (26.7)	0.210
Arthritis, *n* (%)	4 (18.2)	11 (39.3)	0.09
Myocarditis, *n* (%)	2 (9)	0 (0)	0.196
Dyspnoea, *n* %)	13 (59)	9 (32.1)	0.071
Cough, *n* (%)	5 (22.7)	2 (7.1)	0.127
NSIP, *n* (%)	17 (77.3)	15 (53.6)	0.113
UIP, *n* (%)	1 (4.5)	7 (25)	
OP, *n* (%)	4 (18.2)	6 (21.4)	
FVC	92 (71.7–108.5)	79 (69–110)	0.102
TLC	81 (62–101)	79.5 (67.7–89)	0.500
DLCO	69 (59.5–80.5)	70 (51–80)	0.782
KCO	86 (58–98)	87.5 (73.2–109.5)	0.310
CK, U/L	100 (10–181)	107.5 (86.2–483.2)	0.134
CCL18, ng/mL	511 (307–958.7)	207.1 (149.3–381.7)	<0.0001
ANA, *n* (%)	15 (68.2)	19 (67.9)	0.869
Anti-ENA, *n* (%)	15 (68.2)	19 (67.9)	0.981
Myositis-specific antibodies, *n* (%)	19 (86.4)	21 (75)	0.319
Myositis-associated antibodies, *n* (%)	13 (59.1)	15 (53.6)	0.804
Antisynthetase antibodies, *n* (%)	12 (54.5)	16 (57.1)	0.854
Anti-SSA, *n* (%)	11 (50)	11 (39.3)	0.449
Anti-Ro52, *n* (%)	10 (45.5)	10 (35.7)	0.485
Anti-SSB, *n* (%)	2 (9.1)	1 (3.6)	0.415
Anti-Mi2, *n* (%)	1 (4.5)	1 (3.6)	0.861
Anti-U1RNP, *n* (%)	1 (4.5)	1 (3.6)	0.861
Anti-MDA5, *n* (%)	4 (18.2)	2 (7.1)	0.233
Anti-Jo1, *n* (%)	5 (22.7)	12 (42.9)	0.136
Anti-PL12, *n* (%)	4 (18.2)	1 (3.6)	0.087
Anti-PL7, *n* (%)	2 (9.1)	0 (0)	0.103
Anti-PL12 or PL7, *n* (%)	6 (27.3)	1 (3.6)	0.017
Anti-EJ, *n* (%)	2 (9.1)	1 (3.6)	0.415
Anti-Ku, *n* (%)	1 (4.5)	1 (3.6)	0.861
Anti-NXP2, *n* (%)	2 (9.1)	1 (3.6)	0.415
Anti-TIF1γ, *n* (%)	0 (0)	2 (7.1)	0.201
Anti-SRP, *n* (%)	2 (9.1)	0 (0)	0.103
Anti-PMScl, *n* (%)	2 (9.1)	1 (3.3)	0.415
Glucocorticoids, *n* (%)	13 (61.9)	22 (81.5)	0.192
Immunosuppressants, *n* (%)	15 (68.1)	21 (75)	0.276
Mycophenolate, *n* (%)	8 (36.3)	12 (42.8)	0.663
Methotrexate, *n* (%)	4 (18.2)	6 (21.4)	0.972
Azathioprine, *n* (%)	2 (9.1)	1 (3.6)	0.338
Rituximab, *n* (%)	1 (4.5)	1 (3.6)	0.779

Values are expressed as numbers and (%) or median and Q1–Q3 as appropriate. ANA, anti-nuclear antibodies; ASyS, antisynthetase syndrome; CK, creatine kinase; DLCO, diffusion lung CO; DM, dermatomyositis; ENA, extractable nuclear antigen; FVC, forced vital capacity; ILD, interstitial lung disease; KCO, carbon monoxide transfer coefficient; MDA5, anti-melanoma differentiation-associated gene; MMT, manual muscle test; OP, organizing pneumonia; NSIP, nonspecific interstitial pneumonia; PM, polymyositis; RNP, ribonucleoprotein; SRP, signal recognition particle; TIF1γ, transcription intermediary factor 1-gamma; TLC, total lung capacity; UIP, usual interstitial pneumonia.

**Table 4:** Multivariate analysis for the occurrence of ILD progression.

Variable	OR (CI 95%)	*p*
Arthritis	0.357 (0.073–1.759)	0.206
Anti-PL12 or anti-PL7	3.730 (0.288–48.40)	0.314
Immunosuppressants	2.668 (0.429–16.58)	0.293
CCL18 serum levels (ng/mL)	1.006 (1.002–1.011)	0.005

## Data Availability

Not applicable.
